# Human Adenoviruses, Cholesterol Trafficking, and NF-κB Signaling

**Published:** 2018-01-16

**Authors:** Nicholas L. Cianciola, Cathleen R. Carlin

**Affiliations:** 1 Departments of Molecular Biology and Microbiology, School of Medicine, Case Western Reserve University, Cleveland, Ohio 44106; 2 The Case Comprehensive Cancer Center, School of Medicine, Case Western Reserve University, Cleveland, Ohio 44106; 3 The Lockwood Group, Stamford, CT 06901

**Keywords:** Human adenoviruses, cholesterol trafficking, innate immunity, immune evasion

## Abstract

The interplay between viruses and host factors regulating inflammatory or cytotoxic responses directed against infected cells is well documented. Viruses have evolved a wide array of mechanisms that strike a balance between the elimination of virus and immune-mediated tissue injury by antiviral immune responses. The topic of this mini-review is a series of recent studies demonstrating a link between cholesterol trafficking and innate immune responses in cells infected with human adenoviruses that provide the backbone of commonly used vectors in gene medicine. Besides revealing an unexpected role for lipid metabolism in immune evasion, these studies have important implications for understanding the molecular basis of cholesterol trafficking in normal cells and various disease states. They also describe a previously unappreciated host-virus interaction that may be employed by other pathogens to interfere with the host innate immune system.

## Introduction

The NF-κB (nuclear factor kappa-light-chain-enhancer of activated B cells) transcription factor complex is found in almost all animal cells where it regulates the expression of more than 100 target genes^1^. Since many of these genes control the immune response or cell survival, the NF-κB pathway is an attractive target for viral pathogens^2^. The molecular regulation of the NF-κB pathway is well-established. Briefly, IκB inhibits NF-κB gene transcription by sequestering latent NF-κB proteins in the cytoplasm^2^. A diverse array of cytokines and stress-inducing agents trigger signaling pathways that activate IκB kinase (IKK), which subsequently phosphorylates IκB targeting it for ubiquitination and degradation and releasing NF-κB for nuclear translocation and transactivation of NF-κB responsive genes. NF-κB-dependent gene expression is up-regulated by viral products that activate proximal signaling pathways, including the Tat protein of human immunodeficiency virus (HIV-1), the Tax protein of human T-lymphotropic virus (HTLV), and the LMP1 protein of Epstein-Barr virus (EBV)^3^. NF-κB is also activated downstream of protein kinase R (PKR) upon binding of double-stranded RNA replication intermediates employed by RNA viruses^4^. Virus-induced NF-κB activation promotes several functions including viral replication, the host immune response to infection, and protection from virus-induced apoptosis^2^. Other viral gene products, such as the A49 protein of vaccinia virus that blocks IκB ubiquitination and degradation, support immune evasion and virulence by inhibiting NF-κB activation^5^. The NF-κB pathway is also regulated by a dedicated endolysosomal system which controls the signaling output and down-regulation of cell surface receptors that initiate NF-κB activation^6^. The extent to which viruses evade innate immunity by hijacking this pathway represents an important new area of investigation.

## NF-κB and human adenoviruses (HAdVs)

HAdVs are small non-enveloped DNA viruses that provide the backbone for the most widely used viral vectors in gene-based medicine^7^. However, the host innate immune system that activates inflammatory or cytotoxic responses directed against viruses is a major impediment to the efficacy of HAdV vectors^7^. It is well-established that NF-κB has a significant role in the expression of various cytokines and chemokines in cells targeted for HAdV infection, including innate effector cells such as macrophages and non-hematopoietic epithelial and endothelial cells^8^. Although viral cell entry is necessary for this immediate-early response, the coxsackie-adenovirus receptor (CAR), which is the high affinity receptor for all HAdVs except for those belonging to the Group B serotype, does not appear to be directly involved in signal transduction^8,9^. HAdVs do induce several signaling cascades when they engage α_v_-integrins that mediate their uptake via clathrin-coated pits, but none of these pathways have been directly linked to NF-κB activation^8,9^. It has been shown that many HAdVs bind the blood coagulation factor X (FX), and that HAdV-FX complexes activate NF-κB by signaling through Toll-like receptor 4 (TLR4)^10,11^. However, it is unclear if this is the sole means of NF-κB activation or if redundant mechanisms feed into this pathway in HAdV-infected cells.

**Figure 1:**
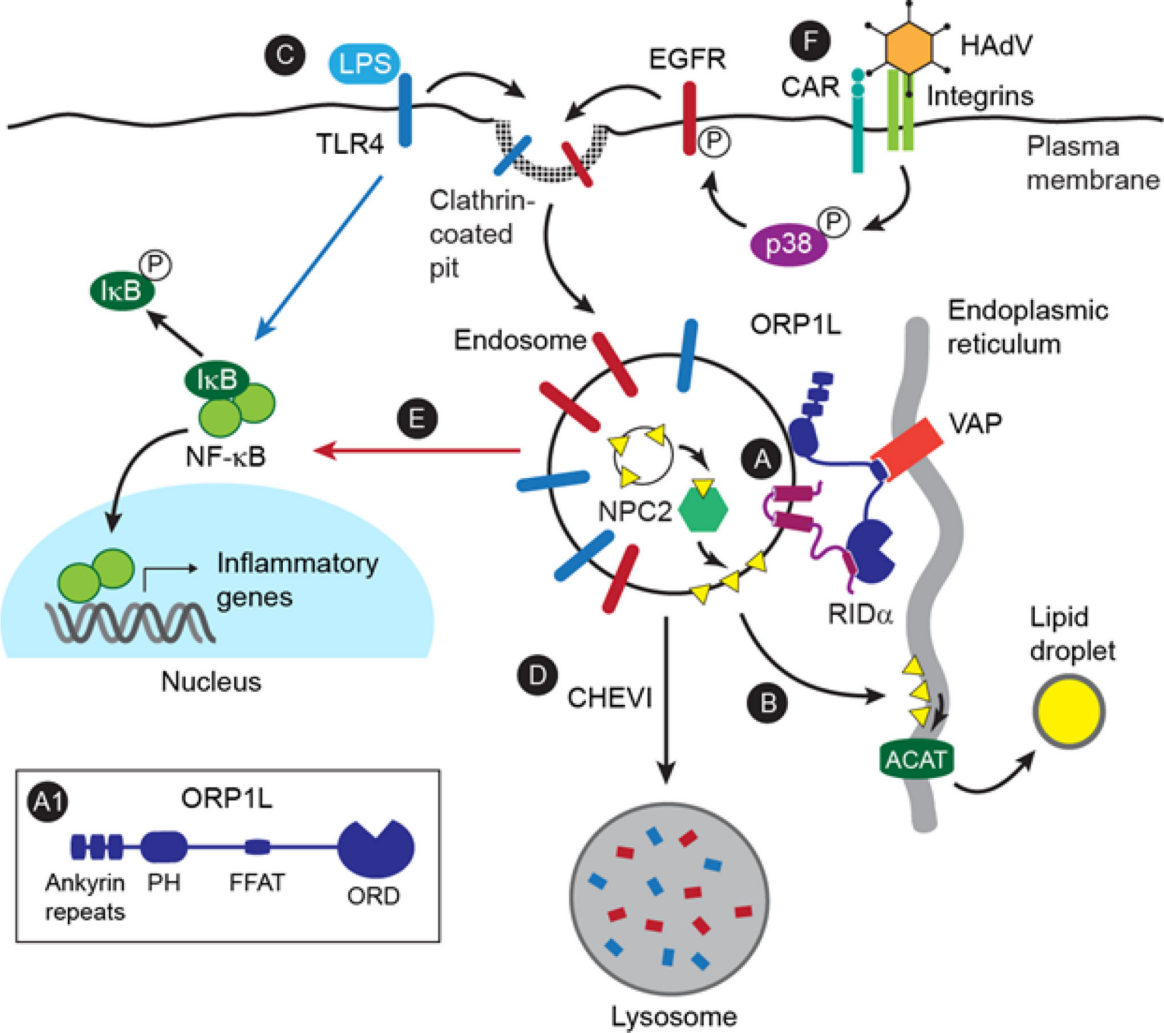
Integrated Model (A) It is established that the interaction between the HAdV protein RIDα and ORP1L stabilizes the interaction between ORP1L and VAP integral membrane proteins forming membrane contacts between endosomes and the ER^14,26^. The ORP1L domain structure described in the text is shown in the inset. (B) It is also established that RIDα-ORP1L complexes support the transport of cholesterol (yellow triangle), which has been transferred from internal membranes to endosomal limiting membranes by NPC2, to ACAT substrate pools in the ER where it is esterified and stored in lipid droplets^14,26^. (C) In addition, it is establshed that RIDα-ORP1L complexes attenuate NF-κB signaling downstream of TLR4 receptors stimulated by LPS^14^. (D) Studies have shown that mutations in proteins comprising the CHEVI tethering complex terminate TLR4 signaling by regulating the clearance of endosomes containing internalized receptors^41^. (E) It is established that EGFRs activate NK-κB signaling from endosomes downstream of p38MAPK signaling independently of extrinsic ligand^51^. (F) We speculate that HAdV cell entry, which is mediated by interactions with CAR and αβ integrins, induces clathrin-mediated EGFR internalization downstream of p38MAPK signaling^9^ leading to NF-κB signaling attenuated by RIDα -regulated cholesterol trafficking.

First-generation HAdV gene therapy vectors were deleted for several early transcription regions (E1A, E1B, and E3) so they were replication-defective and could accommodate carrying foreign genes^12^. These vectors induced high-level innate inflammatory responses within 24 hours of transduction by mechanisms regulated by viral cell entry and expression of early viral gene products^7–9^. Natural Ad infections cause cold-like symptoms but rarely serious illness in immune-competent individuals, suggesting that the early HAdV transcription regions deleted in first generation vectors encoded proteins capable of inhibiting inflammation^7^. Although the first evidence that transcripts from the E3 region blocked NF-κB activation was published nearly two decades ago, the underlying mechanism remained elusive until recently^13^. Studies published in^14^ showed that the E3 protein called “RIDα” attenuated NF-κB activation during an acute infection, and was also sufficient to modulate NF-κB activity downstream of TLR4 signaling independent of infection or expression of other HAdV proteins (Figure 1C). RIDα is a small double-pass intrinsic membrane protein localized to endosomes that lacks intrinsic enzymatic activity and interacts with multiple host proteins regulating endosome function^15–17^. The effect of RIDα on NF-κB signaling was regulated by its ability to bypass cellular machinery facilitating cholesterol trafficking from endosomes to regulatory pools in the endoplasmic reticulum (ER)^14^.

## Cholesterol homeostasis

Cholesterol is critically important for lipid-controlled membrane homeostasis, trafficking, and signaling, and defects in cholesterol trafficking exert widespread effects on cell physiology^18^. In addition to *de novo* synthesis in the ER, cholesterol is taken up in low-density lipoprotein (LDL) particles via receptor-mediated endocytosis^19^. LDL-derived cholesterol is trafficked to other cellular membranes, including the ER where it suppresses activity of sterol regulatory element-binding proteins (SREBP) transcription factors that regulate multiple genes involved in cholesterol and lipid metabolism^20^. Excessive levels of endosomal cholesterol are also converted into cholesteryl esters by acyl-CoA cholesterol acyltransferase (ACAT) in the ER and stored in lipid droplets^21^. LDL-cholesterol trafficking is known to be regulated by NPC1, a large 13-transmembrane protein localized to the limiting membranes of endosomes and lysosomes; and NPC2, a small soluble protein found in the lysosomal lumen^22,23^. Mutations in genes encoding NPC1 or NPC2 cause the lysosomal storage disease Niemann–Pick type C (NPC) characterized by lysosomal accumulation of cholesterol and other lipids on a cellular level, and progressive dementia and death usually before or during adolescence^24^. It has been reported previously that cells infected with an HAdV mutant deleted for RIDα exhibited an NPC-like cholesterol storage phenotype^25^. In addition to preventing an abnormal cholesterol stroage phenotype in infected cells, the RIDα protein was sufficient to restore cholesterol trafficking from endosomes to ACAT substrate pools in the ER in the absence of functional NPC1 protein^25,26^. Sterol transport was manifested by enhanced incorporation of a radioactive LDL-cholesterol pulse into cholesteryl esters accompanied by a dramatic increase in lipid droplet accumulation. In contrast to NPC1, RIDα did not compensate for loss-of-NPC2 function suggesting the viral protein co-opted a key intermediate step coordinating the function of the canonical NPC1/NPC2 machinery. Altogether, these data suggested that RIDα reconstituted cholesterol homeostasis downstream of NPC2 following HAdV infection. Although how HAdV disrupts cholesterol trafficking remains unclear, it is known that the viral particle disrupts the cellular machinery regulating the motility and positioning of NPC1-positive late endosomes and lysosomes in order to facilitate its own transport to the nucleus for replication^27^.

Previous studies showed that the cholesterol trafficking function of the RIDα protein was regulated by its direct interaction with a lipid binding protein called ORP1L belonging to the oxysterol-binding protein related-protein (ORP) family^16,26^ (Figure 1A). In addition to a conserved lipid-binding OSBP-related domain (ORD), ORPs have structural features suggesting they form tethers contributing to the formation of membrane contact sites between adjacent organelles^28^ (Figure 1A.1). In the case of ORP1L, there is a pleckstrin homology (PH) domain that is partially responsible for targeting ORP1L to late endosomes and lysosomes^29^; and an FFAT (two phenylalanines in an acidic tract) motif allowing dynamic interactions between ORP1L and ER vesicle-associated membrane protein-associated proteins (VAPs)^30^. Similar to other ORP family members that exchange lipids over membrane contact sites, the ORP1L-ORD has distinct binding sites for sterol and the phosphoinositide PI(4)P^31^. Prior to our studies with RIDα, however, ORP1L was designated as an endosomal sterol sensor that regulated late endosome/lysosome motility and positioning by controlling the affinity of ORP1L-FFAT for VAP proteins downstream of the small GTPase Rab7^30^. In contrast to this sterol sensing activity, the sterol binding site in the ORP1L-ORD was dispensable for sterol trafficking in the RIDα-induced pathway^26^. In fact, ORP1L-VAP protein complexes were stabilized by the adenoviral RIDα protein under sterol loading conditions that normally impede the interaction with VAP proteins in the Rab7-regulated pathway^30^. Although a role for sterol/PI(4)P exchange at ORP1L-VAP membrane contact sites cannot be excluded, this mechanism is difficult to reconcile with the fact that PI(4)P is a relatively minor lipid outside the secretory pathway^32^. It is probably more likely that RIDα supports cholesterol diffusion down a metabolic gradient created by the rapid conversion of cholesterol to cholesteryl esters by ACAT over endosome-ER membrane contact sites^33^ (Figure 1B). Such a metabolic gradient would be exquisitely sensitive to incrementally small changes in local cholesterol concentrations since ACAT activity is enhanced by substrate binding^34^.

These results provide new insights to the prevailing model for the sequential action of the NPC1 and NPC2 proteins in moving cholesterol out of the late endosomes/lysosomes to the ER^35^. This model postulates that NPC2 delivers cholesterol from internal membranes to endosomal limiting membranes followed by lateral diffusion to NPC1 and efflux to the ER by an unknown mechanism. Studies with the viral protein suggest that NPC1 may act as a sterol sensor that regulates the availability of cholesterol for trafficking to the ER over ORP1L-VAP membrane contacts that are inhibited by high levels of cholesterol on endosomal limiting membranes. The RIDα protein may bypass this regulatory role for NPC1 by a direct protein-protein interaction with ORP1L that alleviates cholesterol inhibition of ORP1L-VAP binding.

## Do HAdVs regulate inflammatory endosome maturation?

How then is RID RIDα -induced cholesterol trafficking linked to the NF-κB pathway? In contrast to the canonical endolysosomal system that terminates signaling by growth factor receptors, inflammatory signaling is regulated by a distinct class of endosomes that are stimulated by pattern recognition receptor (PRR) ligands and microbial pathogens^6,36^. It is therefore conceivable that ORP1L-dependent cholesterol homeostasis controls inflammatory signaling by supporting the functional maturation of these inducible compartments. Fine-tuning cholesterol content could influence local membrane properties of inflammatory endosomes allowing recruitment of effector proteins regulating a rate-limiting step in this inducible endolysosomal pathway. For instance, small Rab GTPases that control discrete steps in membrane trafficking are known to be exquisitely sensitive to cholesterol levels in endocytic organelles^37^. It has also recently emerged that fusion with lysosomes, which is responsible for clearing receptors regulating inflammatory responses, is regulated by a newly identified tethering complex called class C Homologues in Endosome–Vesicle Interaction (CHEVI) containing the Sec1/Munc18 (SM) protein VPS33B^38,39^ (Figure 1D). It is conceivable that local cholesterol content is an important factor in the regulation of CHEVI tethers. Mutations in VPS33B are responsible for arthrogryposis-renal dysfunction-cholestasis (ARC) syndrome, a fatal recessive disorder characterized by trafficking defects in multiple organ systems, persistent infections, and sepsis^40^. Similar to disease-causing VPS33B mutations, a RIDα protein with a mutation that blocked its cholesterol trafficking ability was associated with exaggerated NF-κB responses during acute HAdV infections, and also following TLR4 activation in cells with stable RIDα expression^14,41^. In addition, it is worth noting that the RIDα-ORP1L interaction did not regulate sterol transport to SREBP substrate pools^25,26^. Although ACAT-accessible and SREBP regulatory pools are both associated with smooth ER, recent studies have suggested that these pools are distinct and dissociable based on differences in the kinetic delivery of cholesterol to each of these pools and inhibitor sensitivity^42^. Additional studies are needed to determine whether RIDα co-opts an inducible endosome-ER cholesterol transport pathway supporting inflammatory endosome maturation that is selectively hard-wired to ACAT-accessible pools. The ensuing formation of lipid droplets may also meet specific metabolic demands in cells mounting inflammatory responses.

These findings also raise the possibility that the NPC1–NPC2 core machinery utilizes multiple adaptor proteins that allow development of dynamic physiological responses by facilitating sterol transport to discrete ER regulatory pools. For instance, the pathway revealed by HAdV could regulate the formation of stress-induced lipid droplets that are considered to be hallmarks of tumor aggressiveness and chemotherapeutic resistance in some types of human cancer^43^. Mobilization of lipid droplet cholesteryl esters drives the proliferation of pancreatic cancer cells under cholesterol-restricted conditions, suggesting that therapeutics targeting ORP1L-dependent lipid droplet formation could increase sensitivity to cytotoxic cancer drugs^43^.

## How do HAdVs activate NK-κB prior to viral gene expression?

The RIDα protein may also offer insight to the cellular pathway regulating NF-κB activation induced by acute HAdV infection. RIDα was originally discovered for its ability to specifically down-regulate EGF receptors (EGFRs) from intracellular compartments independent of ligand stimulation or receptor ubiquitination required for targeted EGFR degradation in the canonical endolysosomal system^44–46^. The p38 mitogen-activated kinase (p38-MAPK) and its downstream target MAP kinase-activated protein kinase-2 (MAPKAPK-2) are both known to be rapidly activated by HAdV cell entry^47^. EGFR serine residues 1046/1047 are MAPKAPK-2 substrates, and their phosphorylation is associated with clathrin-mediated uptake to a unique subset of stable endosomes where EGFRs are subsequently activated independently of ligand^48,49^. It has been known for nearly two decades that EGFR regulates NF-κB responses via IKK-regulated IKβ phosphorylation following activation by cognate EGFR ligands^50^. More recently this NF-κB activation pathway has been linked to EGFRs that are activated in endosomes following p38-MAPK mediated EGFR internalization^51^ (Figure 1E). This suggests that HAdV cell entry could trigger EGFR transactivation contributing to early inflammatory responses (Figure 1F), and that RIDα controls the duration of these responses by regulating the maturation of inflammatory endosomes. Given that p38-MAPK activation is a common occurrence associated with pathogen infection, future studies should determine whether other viruses have evolved additional mechanisms that support immune evasion by controlling EGFR signaling from inflammatory endosomes.

## Conclusions and future directions

Characterizing the interaction between HAdVs and host pathways has been a rich resource for discovering previously unappreciated cellular mechanisms and gaining insights to viral pathogenesis^52–55^. This mini-review has summarized recent findings linking membrane contact sites regulating trafficking of endosomal cholesterol to the ER and signaling pathways leading to NK-κB activation in HAdV-infected cells (Figure 1). Analysis of the HAdV RIDα protein suggests that ORP1L may be the missing link regulating cholesterol efflux to ACAT substrate pools in the ER, providing a novel perspective regarding the functional relationship between the NPC1 and NPC2 proteins. These studies have also revealed an unexpected connection between cholesterol homeostasis and the maturation of endosomes regulating NK-κB responses that warrants further investigation. Although the RIDα cholesterol trafficking pathway was discovered in non-immune epithelial cells, it presumably has an important role in immune cells such as macrophages targeted by HAdVs^7,8^. The ability of RIDα to down-tune NF-κB signaling should be beneficial in lessening antiviral innate immune responses to HAdV gene therapy vectors. It will also be important to determine if different viruses co-opt the same pathway of cholesterol trafficking to evade immune detection in the host and if so whether drugs targeting this pathway have broad antiviral properties.

## References

[1] Napetschnig J, Wu H (2013). Molecular Basis of NF-kB Signaling. Annu Rev Biophys.

[2] Hiscott J, Kwon H, Genin P (2001). Hostile takeovers: viral appropriation of the NF-kB pathway. J Clin Invest.

[3] Pahl HL (1999). Activators and target genes of Rel/NF-kB transcription factors. Oncogene.

[4] Garcia MA, Meurs EF, Esteban M (2007). The dsRNA protein kinase PKR: Virus and cell control. Biochimie.

[5] Mansur DS, C Maluquer de Motes L, Unterholzner RP (2013). Poxvirus targeting of E3 ligase beta3-TrCP by molecular mimicry: A mechanism to nhibit NF-kappaB activation and promote immune evasion and virulence. PLOS Pathogens.

[6] Vidya MK, Kumar VG, Sejian V (2017). Toll-like receptors: Significance, ligands, signaling pathways, and functions in mammals. Intl Rev Immunol Oct.

[7] Hendrickx R, Stichling N, Koelen J (2014). Innate immunity to adenovirus. Hum Gene Ther.

[8] Muruve DA (2004). The innate immune response to adenovirus vectors. Hum Gene Therapy.

[9] Wolfrum N, Greber UF (2012). Adenovirus signalling in entry. Cell Micro.

[10] Antoniak S, Mackman N (2014). Multiple roles of the coagulation protease cascade during virus infection. Blood.

[11] Doronin K, Flatt JW, Di Paolo NC (2012). Shayakhmetov Coagulation factor X activates innate immunity to human species C adenovirus. Science.

[12] Danthinne X, Imperiale MJ (2000). Production of first generation adenovirus vectors: a review. Gene Therapy.

[13] Friedman JM, Horwitz MS (2002). Inhibition of Tumor Necrosis Factor Alpha-Induced NF-κB Activation by the Adenovirus E3-10.4/14.5K Complex. J Virol.

[14] Cianciola NL, Chung S, Manor D (2017). Adenovirus modulates Toll-like receptor 4 signaling by reprogramming ORP1L-VAP protein contacts for cholesterol transport from endosomes to the endoplasmic reticulum. J Virol.

[15] Hoffman P, Yaffe MB, Hoffman BL (1992). Characterization of the adenovirus E3 protein that down-regulates the epidermal growth factor receptor. J Biol Chem.

[16] Shah AH, Cianciola NL, Mills JL (2007). Adenovirus RIDa regulates endosome maturation by mimicking GTP-Rab7. J Cell Biol.

[17] Cianciola NL, Crooks D, Shah AH (2007). A tyrosine-based signal plays a critical role in the targeting and function of adenovirus RID{alpha} protein. J Virol.

[18] Ikonen E (2008). Cellular cholesterol trafficking and compartmentalization. Nat Rev Mol Cell Biol.

[19] Brown M, Goldstein J (1986). A receptor-mediated pathway for cholesterol homeostasis. Science.

[20] Brown MS, Goldstein JL (1997). The SREBP pathway: Regulation of cholesterol metabolism by proteolysis of a membrane-bound transcription factor. Cell.

[21] Chang TY, Chang CCY, Ohgami N (2006). Cholesterol sensing, trafficking, and esterification. Annu Rev Cell Dev Biol.

[22] Neufeld EB, Wastney M, Patel S (1999). The Niemann-Pick C1 protein resides in a vesicular compartment linked to retrograde transport of multiple lysosomal cargo. J Biol Chem.

[23] Naureckiene S, Sleat DE, Lackland H (2000). Identification of HE1 as the second gene of Niemann-Pick C disease. Science.

[24] Patterson MC, Vanier MT, Suzuki K, Scriver CR, Beaudet AL, Sly WS, Valle D (2001). Niemann Pick Disease Type C: A Lipid Trafficking Disorder. The Metabolic and Molecular Bases of Inherited Disease.

[25] Cianciola NL, Carlin CR (2009). Adenovirus RID-a activates an autonomous cholesterol regulatory mechanism that rescues defects linked to Niemann-Pick disease type C. J Cell Biol.

[26] Cianciola NL, Greene DJ, Morton RE (2013). Adenovirus RIDa uncovers a novel pathway requiring ORP1L for lipid droplet formation independent of NPC1. Mol Biol Cell.

[27] Scherer J, Yi J, Vallee RB (2014). PKA-dependent dynein switching from lysosomes to adenovirus: A novel form of host-virus competition. J Cell Biol.

[28] Suchanek M, Hynynen R, Wohlfahrt G (2007). The mammalian oxysterol-binding protein-related proteins (ORPs) bind 25-hydroxycholesterol in an evolutionarily conserved pocket. Biochem J.

[29] Johansson M, Lehto M, Tanhuanpaa K (2005). The oxysterol-binding protein homologue ORP1L interacts with Rab7 and alters functional properties of late endocytic compartments. Mol Biol Cell.

[30] Rocha N, Kuijl C, van der Kant R (2009). Cholesterol sensor ORP1L contacts the ER protein VAP to control Rab7-RILP-p150Glued and late endosome positioning. J Cell Biol.

[31] Vihervaara T, Uronen RL, Wohlfahrt G (2011). Sterol binding by OSBP-related protein 1L regulates late endosome motility and function. Cell Mol Life Sci.

[32] Choudhury R, Hyvola N, Lowe M (2005). Phosphoinositides and membrane traffic at the trans-Golgi network. Biochem Soc Symp.

[33] Quon E, Beh CT (2015). Membrane contact sites: Complex zones for membrane association and lipid exchange. Lipid Insights.

[34] Chang CCY, Lee CYG, Chang ET (1998). Recombinant acyl-CoA:cholesterol acyltransferase-1 (ACAT-1) purified to essential homogeneity utilizes cholesterol in mixed micelles or in vesicles in a highly cooperative manner. J Biol Chem.

[35] Vance JE (2010). Transfer of cholesterol by the NPC team. Cell Metab.

[36] Kinchen JM, Doukoumetzidis K, Almendinger J (2008). A pathway for phagosome maturation during engulfment of apoptotic cells. Nat Cell Biol.

[37] Simons K, Gruenberg J (2000). Jamming the endosomal system: lipid rafts and lysosomal storage diseases. Trends in Cell Biology.

[38] Spang A (2016). Membrane tethering complexes in the endosomal system. Frontiers Cell Dev Biol.

[39] Rogerson C, Gissen P (2016). The CHEVI tethering complex: facilitating special deliveries. J Pathol.

[40] Jang JY, Kim KM, Kim GH (2009). Clinical characteristics and VPS33B mutations in patients with ARC syndrome. J Pediatr Gastroenterol Nutr.

[41] Akbar MA, Mandraju R, Tracy C (2016). ARC syndrome-linked Vps33B protein is required for inflammatory endosomal maturation and signal termination. Immunity.

[42] Kristiana I, Yang H, Brown AJ (2008). Different kinetics of cholesterol delivery to components of the cholesterol homeostatic machinery: Implications for cholesterol trafficking to the endoplasmic reticulum. Biochim Biophys Acta - Mol Cell Biol Lipids.

[43] Beloribi-Djefaflia S, Vasseur S, Guillaumond F (2016). Lipid metabolic reprogramming in cancer cells. Oncogenesis.

[44] Hoffman P, Carlin C (1994). Adenovirus E3 protein causes constitutively internalized EGF receptors to accumulate in a prelysosomal compartment, resulting in enhanced degradation. Mol Cell Biol.

[45] Tsacoumangos A, Kil SJ, Ma L (2005). A novel dileucine lysosomal-sorting-signal mediates intracellular EGF-receptor retention independently of protein ubiquitylation. J Cell Sci.

[46] Hoffman PH, Rajakumar P, Hoffman B (1992). Evidence for intracellular down-regulation of the epidermal growth factor receptor during adenovirus infection by an EGF-independent mechanism. J Virol.

[47] Suomalainen M, Nakano MY, Boucke K (2001). Adenovirus-activated PKA and p38/MAPK pathways boost microtubule-mediated nuclear targeting of virus. EMBO J.

[48] Tan X, Lambert PF, Rapraeger AC (2016). Stress-induced EGFR trafficking: mechanisms, functions, and therapeutic implications. Trends Cell Biol.

[49] Tomas A, Futter CE, Eden ER (2014). EGF receptor trafficking: consequences for signaling and cancer. Trends Cell Biol.

[50] Shostak K, Chariot A (2015). EGFR and NF-kB: partners in cancer. Trends Mol Med.

[51] El-Hashim AZ, Khajah MA, Renno WM (2017). Src-dependent EGFR transactivation regulates lung inflammation via downstream signaling involving ERK1/2, PI3K/Akt and NF-kB induction in a murine asthma model. Sci Rep.

[52] Duffy MR, Parker AL, Bradshaw AC (2012). Manipulation of adenovirus interactions with host factors for gene therapy applications. Nanomedicine.

[53] Kremer EJ, Nemerow GR (2015). Adenovirus tales: From the cell surface to the nuclear pore complex. PLOS Pathogens.

[54] Windheim M, Hilgendorf A, Burgert H (2004). Immune evasion by adenovirus E3 proteins: exploitation of intracellular trafficking pathways. Curr Top Microbiol Immunol.

[55] Alto NM, Orth K Subversion of cell signaling by pathogens. CSH Perspectives Biol.

